# Micromolar affinity CAR T cells to ICAM-1 achieves rapid tumor elimination while avoiding systemic toxicity

**DOI:** 10.1038/s41598-017-14749-3

**Published:** 2017-10-30

**Authors:** Spencer Park, Enda Shevlin, Yogindra Vedvyas, Marjan Zaman, Susan Park, Yen-Michael S. Hsu, Irene M. Min, Moonsoo M. Jin

**Affiliations:** 1 Molecular Imaging Innovations Institute, Department of Radiology, Weill Cornell Medicine, New York, NY 10065 USA; 2 Department of Surgery, Weill Cornell Medicine, New York, NY 10065 USA; 3 Department of Pathology and Laboratory Medicine, Weill Cornell Medicine, New York, NY 10065 USA; 4 Department of Biomedical Engineering, Cornell University, Ithaca, NY 14850 USA

## Abstract

Adoptive transfer of high-affinity chimeric antigen receptor (CAR) T cells targeting hematological cancers has yielded impressive clinical results. However, safety concerns regarding target expression on healthy tissue and poor efficacy have hampered application to solid tumors. Here, a panel of affinity-variant CARs were constructed targeting overexpressed ICAM-1, a broad tumor biomarker, using its physiological ligand, LFA-1. Anti-tumor T cell potency *in vitro* was directly proportional to CAR affinity and ICAM-1 density. In a solid tumor mouse model allowing simultaneous monitoring of anti-tumor potency and systemic off-tumor toxicity, micromolar affinity CAR T cells demonstrated superior anti-tumor efficacy and safety compared to their nanomolar counterparts. Longitudinal T cell tracking by PET/CT and concurrent cytokine measurement revealed superior expansion and contraction kinetics of micromolar affinity CAR T cells. Therefore, we developed an ICAM-1 specific CAR with broad anti-tumor applicability that utilized a reduced affinity targeting strategy to significantly boost efficacy and safety.

## Introduction

CAR molecules are composed of synthetic binding moieties, typically an antibody-derived single chain fragment variable (scFv) or any native antigen-sensing element, fused to intracellular signaling domains composed of the T cell receptor (TCR) zeta chain and costimulatory molecules such as CD28 and/or 4-1BB^1,2^. The advantages of CAR mediated targeting include: (1) the provision of activation, proliferation, and survival signals in-cis via a single binding event, compared to the natural, non-integrated TCR and costimulatory signaling; (2) the ability to bypass the downregulation of major histocompatibility complex (MHC) by tumor cells through MHC-independent antigen recognition; and (3) a reduced activation threshold as well as recognition of tumor cells with low antigen density enabled by the high affinity interaction between CAR and antigen^3,4^. As such, T cells modified with scFv-based CARs specific for the pan B cell antigen CD19 have demonstrated unprecedented remission rates in relapsed and refractory B cell leukemia and lymphomas^5–8^. However, CD19 CAR T cell therapies have also caused profound treatment-related toxicities, such as cytokine release syndrome, encephalopathy, B cell aplasia, and coagulopathy^9^. In comparison, the advancement of CAR T cell therapy in solid tumors has been limited due to the scarcity of tumor antigens that are deemed safe for targeting. Thus far, clinical outcomes in solid malignancies have been poor in comparison to those in hematological settings^10,11^, and methods to improve efficacy are being actively investigated.

The ideal CAR target antigen would be a native, surface-exposed tumor neoantigen that is highly expressed and is undetectable in healthy tissues. However, due to the implicit rarity of such antigens, many commonly targeted solid tumor antigens, such as human epidermal growth factor receptor 2 (ErbB2), epidermal growth factor receptor (EGFR), mucin 1 (MUC1), prostate-specific membrane antigen (PSMA), and disialoganglioside (GD2)^10^, are also expressed by non-tumor tissues, albeit at lower levels. CAR molecules with high affinity to such antigens can lead to collateral targeting of healthy tissues resulting in on-target, off-tumor toxicity, a major limiting factor to the progress of CAR T cell therapy to date. In the case of CD19-specific CAR T cells, elimination of healthy B cells is a manageable morbidity and therefore has not been a critical safety issue. However, recent reports on severe adverse toxicities and fatalities associated with CAR T cells in solid tumor settings^12–14^ illustrate the importance of ligand-receptor pair selection and the role of affinity in determining the therapeutic index.

The affinity of a TCR for its cognate peptide-MHC (pMHC) typically ranges between 1–100 μM, thus endowing T cells with tolerance towards cells with subthreshold levels of pMHC expression^15–17^. Similarly, T cells possessing micromolar affinity (1 μM) CARs are capable of lysing cells overexpressing target antigens while sparing those with much lower densities^18^. The affinity and avidity of a CAR for its target antigen also influences T cell cytokine release, the rate of tumor killing, and T cell persistence^3,18–20^. Studies using engineered TCRs with pMHC affinities significantly above their natural range caused T cells to exhibit rapid exhaustion and poor persistence *in vivo* ^21–23^. Together, these studies emphasize the need to reconsider the criteria used to determine optimal CAR affinities to achieve enhanced therapeutic indices.

In this study, we redirected affinity variant CAR T cells toward intercellular adhesion molecule (ICAM)-1, a molecule that is upregulated in several carcinomas and the associated stroma^24^ as well as in inflammatory conditions^25^. Aside from diseased tissues, ICAM-1 is basally expressed in several cell types including endothelial cells, immune cells, and some epithelial cells^25^. The antigen-binding domain of the CAR used in this study is constructed from the native ligand for ICAM-1, the inserted (I) domain of lymphocyte function-associated antigen (LFA)-1, which exhibits a solution affinity of approximately 1 mM^26^. Previously, the I domain was engineered using directed evolution to isolate variants exhibiting affinities to ICAM-1 that span six orders of magnitude (K_D_ ~1 nM to 1 mM)^27^. As human LFA-1 I domain exhibits reactivity with both murine and human ICAM-1^28^ at comparable affinities, we were able to preclinically address potency against human tumors overexpressing ICAM-1 and simultaneously monitor potential on-target, off-tumor systemic toxicity against normal mouse tissues expressing murine ICAM-1. We found that I domain CARs exhibiting affinities in the micromolar range (~10 μM) exhibited a significantly higher therapeutic index *in vivo* compared to CARs with higher affinity (1–100 nM) which tended to cause unbiased reactivity against normal cells with basal ICAM-1 expression and led to less efficient tumor regression. Simultaneous expression of a reporter gene, human somatostatin receptor 2 (SSTR2), on affinity-variant CAR T cells, enabled longitudinal, positron emission tomography and computed tomography (PET/CT)-based spatiotemporal mapping of adoptively transferred T cells in real time^29^. This provided a unique additional insight into the dynamics of CAR T cell behavior *in vivo* which both substantiated and expanded upon observations made using traditional methodologies, demonstrating for example, the exact location of a potentially lethal occurrence of on-target, off-tumor CAR T cell expansion.

## Experimental Procedures

### Cell lines and primary human lymphocytes

Human dermal microvascular endothelial cells (HMEC-1) were obtained from the Center for Disease Control and were cultured in MCDB 131 medium (Invitrogen) supplemented with 10% (v/v) fetal bovine serum (FBS, Atlanta Biologicals), 10 mM L-alanyl-L-glutamine dipeptide (Gibco), 100 units/ml Penicillin-Streptomycin (Pen-strep), 1 μg/ml hydrocortisome (MP Biomedicals), and 10 ng/ml recombinant human epidermal growth factors (Invitrogen). Mouse brain microvascular endothelial cells (bEnd.3, ATCC) were maintained in Advanced Dulbecco’s Modified Eagle Medium (ADMEM, Invitrogen) supplemented with 4 mM L-glutamine, 100 units/ml Pen-strep, and 10% FBS. HeLa cells (ATCC) were cultured in ADMEM containing 10% FBS, 2 mM L-glutamine, and 100 units/ml Pen-strep. 8505 C cells (DSMZ) were cultured in RPMI-1640 medium (Invitrogen) containing 10% FBS, 2 mM L-glutamine, and 100 units/ml Pen-strep. HMEC-1, bEnd.3, HeLa, and 8505 C cells were transduced with lentivirus encoding Firefly Luciferase-F2A-GFP (Biosettia) and sorted based on fluorescence.

Human peripheral blood was obtained from healthy volunteer donors by venipuncture. Peripheral blood mononuclear cells were isolated using Ficoll-Paque PLUS (GE Healthcare) and cultured in Optimizer CTS T-cell Expansion SFM (Thermo) supplemented with 5% human AB serum (Sigma), 2 mM L-alanyl-L-glutamine dipeptide, and 30 IU/ml human IL-2 (Cell Sciences) (T cell culture medium). Non-adherent cells were removed after 24 h and enriched for T cells with Dynabeads CD3/CD28 T cell expander (Thermo) at a 2:1 bead to T cell ratio. Dynabead-bound T cells were subsequently cultured in IL-2 containing media at a density of 1 × 10^6^ cells/ml. All cells were incubated at 37 °C in a 5% CO_2_ humidified incubator.

### Construction of I domain CAR vector

Genetic sequences encoding LFA-1 I domains of varying affinities to ICAM-1 were derived from a previous study^27^. I domain variants were fused at the C-terminus directly to the CD8 hinge, CD28 transmembrane domain, and the intracellular portions of the 3^rd^ generation CAR architecture incorporating the cytoplasmic domains of CD28, CD137, and CD3ζ. The complete CAR inserts were then subcloned into a pLenti backbone^29^. A reporter gene for CAR T cell imaging, SSTR2, was linked to I domain at the N-terminus using a ‘ribosome skipping’ porcine teschovirus-1 2 A (P2A) sequence to ensure comparable production of CAR and SSTR2 from the same mRNA.

### Lentivirus production and transduction of T cells

Lentivirus was produced by transiently transfecting HEK 293 T cells using calcium phosphate. Briefly, 10 µg of transfer gene, 7.5 µg of pCMV-dR8.2 (Addgene) and 5 µg of pCMV-VSVG (Addgene) were mixed and incubated with 2 M CaCl_2_ followed by 2x HBSS. Resulting solutions were added dropwise to 10 cm^2^ cell culture dishes seeded with 3.2 × 10^6^ HEK 293 T cells in 10 ml DMEM 24 h previously. Transfection media was replaced after 6 h. Media containing lentivirus was harvested at 48 and 72 h post transfection, filtered through 0.45 µm filters, and concentrated by ultracentrifugation at 75,000x g for 2 h at 4 °C. Lentivirus was then resuspended in serum containing media and frozen at −80 °C. Human T cells were transduced 24–72 h post activation with anti-CD3/CD28 Dynabeads either by spinfection (1,000 g for 1 h at 32 °C) or by overnight incubation with lentivirus. T cells were transduced once more 24 h after the first transduction. During and following transductions, media containing IL-2 was replaced with media containing human IL-7 (10 ng/ml) and IL-15 (5 ng/ml) (Peprotech). Jurkat T cells were transduced by a single overnight incubation with lentivirus.

### *In vitro* target cell killing assay

2 × 10^5^ target cells (HMEC-1, bEnd.3, HeLa, and 8505c) stably transduced to express GFP and firefly luciferase were co-cultured with either non-transduced or transduced T cells at varying effector to target ratios (E:T). The number of effector T cells was the total number of T cells without accounting for the difference in the level of transduction. In certain conditions, the ICAM-1 gene was disrupted in 8505 C cells using CRISPR/Cas9 (Santa Cruz, #sc-400098; denoted as 8505 C/-ICAM-1) or alternatively, 8505 C cells were exposed to 1 μg/ml lipopolysaccharide (LPS; *Escherichia coli* O26:B6, Sigma) for 12 h to induce overexpression of ICAM-1 (denoted as 8505 C/LPS). Co-cultures were carried out in T cell culture medium containing 150 µg/ml D-Luciferin (Gold Biotechnology) and no cytokine supplementation. Luminescence was measured using a plate reader (TECAN infinite M1000 PRO) with readings in each E:T condition normalized to the non-transduced T cell:target co-culture controls.

### 8505 C mouse model, whole-body tumor imaging, and serum cytokine analysis

7.5 × 10^5^ 8505 C cells were injected into non-obese diabetic/LtSz-*Prkdc* ^*scid*^ *Il2rg* ^*tm1Wjl*^/J (NSG) mice (Jackson Laboratory) via tail vein. 1–3 × 10^6^ T cells were injected via tail vein 8–10 days after tumor cell injection. Injection timing was chosen based on prior studies with R6.5 CAR T cells which demonstrated tumor elimination using similar CAR dosages at up to 10-days post xenograft^29^. Luminescence imaging of tumor xenografts in live mice was performed using a whole body optical imager (*In-Vivo* Extreme, Bruker). Mice were anesthetized with 2% isoflurane in 2 L/min O_2_. Tumor burden was quantified through integration of luminescence over chest cavity and the entire mouse body. For serum cytokine analysis, 50–100 μl of blood was collected via tail-vein into Eppendorf tubes on ice. Plasma was immediately isolated after removing the cell pellet by centrifugation at 2,000 g for 10 min at 4 °C, and stored at −80 °C. Human cytokines (GM-CSF, IL-2, IL-6, IFN-γ, TNF-α, CXCL10) were measured in duplicate using Bio-Plex MAGPIX (Bio Rad) according to the manufacturer’s instructions. In the survival study, mice were euthanized if they exhibited greater than 20–30% loss of body weight accompanied by other signs of severe illnesses (e.g., lethargy, hunched posture, and inactivity).

### *Ex vivo* cellular analysis

Tumor xenografts were resected from mice at appropriate time points. Resected tumors were diced and flushed through 80 µm cell strainers to yield single cell suspensions. Red blood cells were lysed by incubation with 1x RBC lysis buffer (eBiosciences). Remaining cells were washed, re-suspended in 1x HBSS containing 2% normal goat serum, and blocked with mouse IgG at 2 µg/ml for 10 min. This was followed by staining with 1 µg/ml Propidium Iodide (Invitrogen) in combination with 2 µg/ml mouse anti-human CD3-Alexa Fluor 647 (Biolegend) or 2 µg/ml rabbit anti-c-myc-Alexa Fluor 647 (Biolegend). Resulting cells were acquired on a Gallios flow cytometer (Beckman Coulter). Initial flow cytometry gates were determined based on live cell gating (Propidium Iodide negative).

### ICAM-1 and CAR expression quantification

ICAM-1 expression on various cell lines was determined using a mouse anti-human R6.5 monoclonal antibody (10 µg/ml) obtained from hybridoma (ATCC). I domain CAR expression on T cells was detected using 2 µg/ml rabbit anti-c-myc-Alexa Fluor 647 (Biolegend). I domain Jurkat T cell variants were incubated with 10 µg/ml recombinant human ICAM-1 fused to human Fcγ (R&D Systems). Cells were then washed and resuspended in 1 μg/ml rabbit anti-human PE (Santa Cruz Biotechnology) prior to flow cytometry analysis.

### *In vitro* measurement of IFN-γ

Target cells were washed and suspended at 1 × 10^6^ cells/ml in T cell culture medium without cytokines. 100 μl of each target cell suspension was added in triplicate to a 96-well round bottom plate (Corning). T cells suspended at 5 × 10^6^ cells/ml in T cell culture medium were combined with target cells in appropriate wells. Plates were incubated at 37 °C for 24–48 h. After incubation, supernatants were collected for IFN-γ detection by ELISA(Biolegend).

### CD25 and CD69 staNining

Jurkat cells modified with I domain CARs were co-cultured with target cells at an effector to target ratio of 1:1 (1 × 10^5^ effectors: 1 × 10^5^ targets) in a 96-well plate. The plate was incubated at 37 °C for 6 h. After incubation, cells were washed prior to labelling with 2 μg/ml anti-human CD25-allophycocyanin (APC; Biolegend) for 30 min on ice. After incubation, samples were washed and analyzed by flow cytometry. As an alternative to ICAM-1 expressing cells, we also used microbeads coated with known amounts of ICAM-1. 1 × 10^6^ sulfate latex microbeads (8 μm, ThermoFisher Scientific) were resuspended in 100 μL of PBS containing indicated amounts of human or murine recombinant ICAM-1-Fcγ (R&D Systems) conjugated with Cy5.5 (Sulfo-Cyanine5.5 NHS ester, Lumiprobe) overnight at room temperature with gentle mixing. Protein-labeled particles were pelleted and resuspended in fresh PBS containing 0.1 M glycine pH 7.4 for 1 h, while supernatant was used to measure bead adsorption efficiency by fluorescence (TECAN infinite M1000 PRO). After saturation of the bead surface with glycine, beads were pelleted and resuspended in PBS containing 5 mM MgCl_2_. Jurkat cells modified to express each I domain CAR variant were incubated with ICAM-1-bound latex beads at 1:3 (cell:bead) ratio overnight at 37 °C. Cells were then collected and labeled with 2 μg/ml anti-human CD69-APC (Biolegend) for analysis by flow cytometry.

### V-bottom adhesion assay

V-bottom 96-well plates (Corning) were coated with either murine or human ICAM-1-Fcγ (10 μg/ml in PBS, pH 7.4) or 2% BSA at 4 °C overnight. The plates were then blocked with 2% BSA for 1 h at 37 °C. I domain CAR T clones were first stained with CellTracker Orange according to manufacturer’s protocol and then added to ICAM-1-coated wells in 50 μl of PBS containing 5 mM MgCl_2_ and 1% BSA. Plates were immediately centrifuged at 200 *g* for 15 min at room temperature. Nonadherent cells that accumulated at the bottom of the V-bottom plates were quantified by a fluorescence plate reader (TECAN infinite M1000 PRO). Cell binding to ICAM-1 was calculated from the fluorescence intensity values of experimental measurements (F_CAR_ and F_NT_) and normalized to the fluorescence from the wells coated with BSA alone (F_BSA_): 100 × ((F_BSA_ − F_CAR_)/F_BSA_)/((F_BSA_ − F_NT_)/F_BSA_).

### Labeling of ^18^F-NOTA-octreotide (NOTAOCT)

NOTAOCT (1,4,7-Triazacyclononane-1,4,7-triacetic acid-octreotide^30^, GMP grade) was obtained as a 1 mg lyophilized powder (cat #9762, ABX Pharmaceuticals). The NOTAOCT vial content was diluted with 18 MOHM-cm water to 200 μl (5 mg/ml solution) and stored at 4 °C as a stock solution. For chelation of NOTA with Fluorine-18^31^, 5 μl of NOTAOCT was added to 10 μl of 0.1 M sodium acetate, pH 4, 6 μl of 2 mM AlCl3, and 100 μl containing ~30 mCi^18^F. The solution was immediately placed in a Thermomixer (Eppendorf) at 100 °C and incubated for 15 minutes followed by cooling to room temperature and dilution in 15 ml ddH_2_O. A Sep-Pak light C18 column was regenerated in 3 ml 100% ethanol and washed twice in 5 ml ddH_2_O with an observed flow rate of 10 drops per minute. NOTAOCT was then loaded to the Sep-Pak column, which was later washed in 15 ml 18 MW water to eliminate any remaining free^18^F. Trapped NOTAOCT was eluted from the column using 300 μl of ethanol and diluted to 1.5 ml with PBS for injection, providing the final product in ~15% ethanol isotonic, injectable solution. The eluent was passed through 0.2 μm filter. The purity of the final product was checked by reverse phase HPLC.

### PET/CT imaging

Registered CT images were acquired using a micro-PET/CT scanner (Inveon, Siemens) at 1–2 h post NOTAOCT injection. Projection data was acquired in a cone-beam geometry with approximately 1 s steps at 1 degree angular increments. At least 10 million coincidence events were acquired for PET per study using a 250 to 750 keV energy window and a 6 ns timing window. A reference tube containing 100 µl of a 10%ID/cm^3^ equivalent dose for quantification of NOTATOC uptake *in vivo*. To compute NOTAOCT uptake within mouse lungs, ellipsoids were drawn separately on the left and right sides of lungs to enclose the majority of their footprint. The %ID/cm^3^ values, computed relative to the counts obtained in the reference tube, were approximated to a standard uptake value (SUV^32^) by dividing %ID/cm^3^ by four, assuming injection efficiency of 100% and 25 g of body weight. Visualization and analyses of PET/CT images were performed using AMIDE software (http://amide.sourceforge.net).

### Histology

After euthanasia, mouse lungs were perfused via the trachea with 4% paraformaldehyde, and each of five lobes were separated post fixation and embedded in paraffin. Tissues were cut to produce 5 µm sections (Microtome, Leica). Paraffin-embedded sections were stained with hematoxylin and eosin (H&E) or hematoxylin only for CD3 and GFP immunostaining (performed by HistoWiz, Inc.). Histological analysis was performed by an experienced pathologist.

### Statistical analysis

One-way ANOVA, Dunnett’s multiple comparisons test, and unpaired Student’s t-test were performed using Prism (GraphPad) on data indicated.

### Study Approval

The protocol for blood draw along with acquisition of informed consent from healthy volunteers was approved by the Institutional Review Board (IRB) of Weill Cornell Medicine (Permit Number: 1302013613). Studies involving human blood were carried out in strict accordance with Weill Cornell’s IRB guidelines and regulation. All animal experiments were performed in strict accordance with the recommendations contained within the National Institute of Health’s Guide for the Care and Use of Laboratory Animals. Animal handling protocols were approved by the Institutional Laboratory Animal Use and Care Committee of Weill Cornell Medicine (Permit Number: 2012–0063).

## Results

### ICAM-1 specific CAR T cells with 10^6^-fold, step-wise variation in affinity

**Figure 1:**
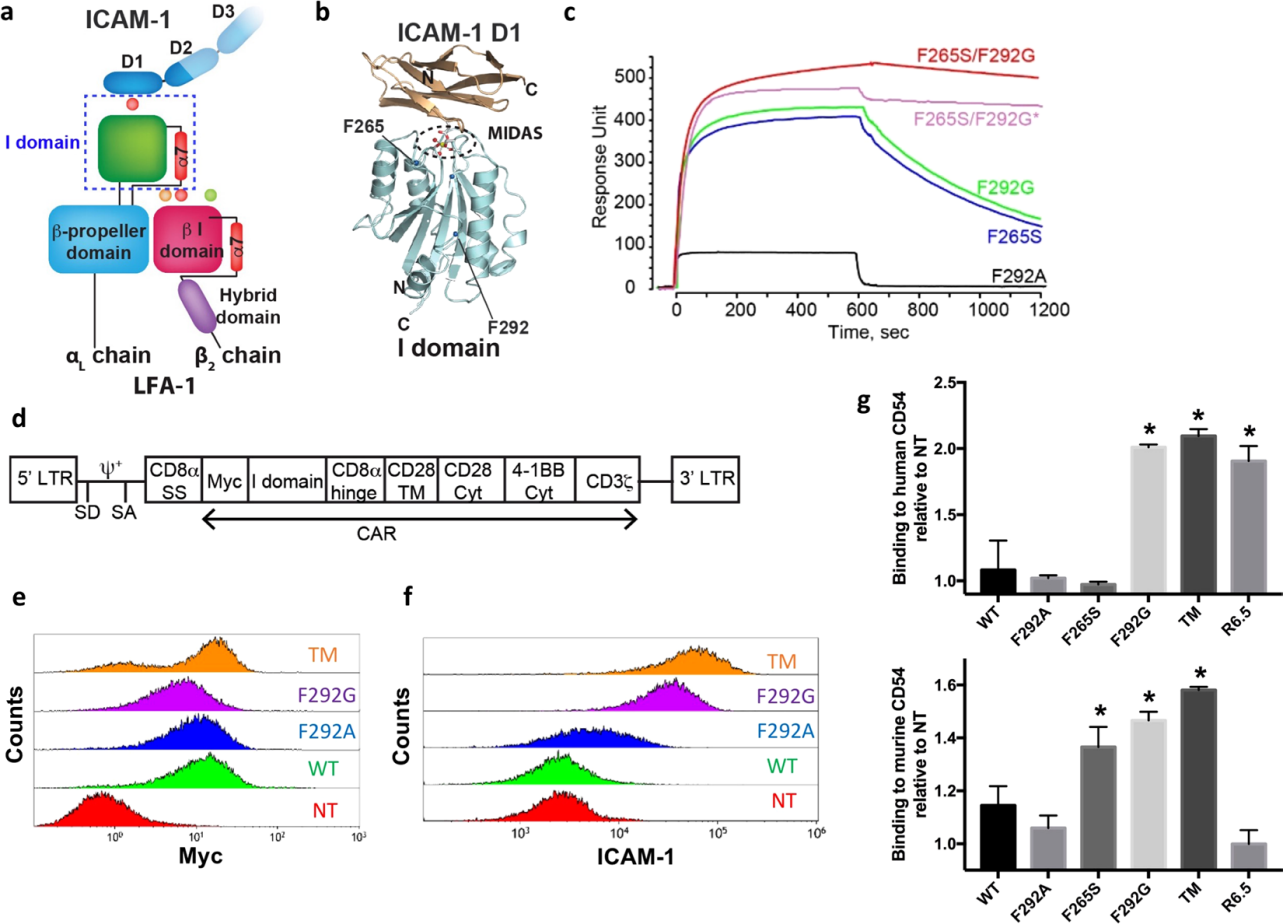
Construction of ICAM-1 specific CARs with step-wise, 10^6^-fold variations in affinity. (**a**) Schematic of LFA-1 in complex with ICAM-1. α and β chains, and modular domains of LFA-1 integrin are labeled. I domain of α chain is denoted with a dotted box. Metal ions necessary for LFA-1 and ICAM-1 interaction are shown in circles. (**b**) Structural model of LFA-1 I domain and the N-terminal domain of ICAM-1 (D1) are drawn in ribbon diagram. N and C-termini, and mutational hot spots are indicated. (**c**) SPR sensogram of I domain variants binding to immobilized human ICAM-1, except F265S/F292G*, which was flowed over murine ICAM-1 (adapted from Fig. 2 of Jin *et al*.^27^, and Fig. 1 of Wong *et al*.^28^). (**d**) A schematic of the lentivirus vector encoding I domain CAR. LTR = long terminal repeat; SD = splice donor; SA = splice acceptor; ψ^+^  = packaging signal; SS = signal sequence; TM = transmembrane; Cyt = cytosolic domain. (**e**) Anti-Myc antibody binding to Jurkat T cells transduced with Myc-tagged CARs (TM, F292G, F292A, and WT I domain). NT = non-transduced. (**f**) Recombinant ICAM-1-Fc binding to CARs expressed in HEK 293 T cells. (**g**) V-bottom adhesion assay measuring relative binding affinities between I domain CARs expressed in Jurkat T cells and soluble human (top) and murine (bottom) ICAM-1 (CD54) coated surfaces. n = 3; *p* < *0*.*01* for * vs. NT by Dunnett’s multiple comparisons test.

CAR constructs specific to ICAM-1 were built using the I domain derived from LFA-1 (Fig. 1a,b). Various activating point mutations in the I domain have previously been isolated, which were localized outside of the binding interface that includes a region known as the metal-ion dependent adhesion site (MIDAS) (Fig. 1b). The step-wise elevation of affinity to ICAM-1 is proposed to be induced by lowering the energy barrier for transition between the inactive (closed) to active (open) I domain conformations. For example, substitution of Phe-292 located in the C-terminal α7-helix with Ala (F292A) and Gly (F292G) led to affinities (K_D_) of ~20 µM and 0.1 µM, respectively (Table 1). The combination of F292G with another comparably activating mutation in Phe-265 (F265S/F292G) led to an affinity of 6 nM, approximately 200,000-fold higher than the wild-type (WT) I domain (K_D_ = 1.5 mM) (Fig. 1c). To lock the C-terminal α7-helix of F265S/F292G in the open position (Fig. 1a), Gly-311 was replaced with Cys (G311C) in the F265S/F292G mutant (F265S/F292G/G311C, dubbed triple mutant or TM) to form a disulfide bond with the naturally unpaired Cys-125 (Table 1). Therefore, the monovalent affinities of individual I domain variants for ICAM-1 span approximately six orders of magnitude (K_D_ ~1 nM to 1 mM), as measured by surface plasmon resonance (SPR) or estimated by flow cytometry (Fig. 1c; Table 1). Human I domain binds murine ICAM-1 with comparable affinity to human ICAM-1 (2 nM vs. 6 nM respectively; Table 1. Similar observations using mutated LFA-1 have been reported by others^26^). Cross-reactivity with its murine homologue enables examination of on-target, off-tumor toxicity of I domain CAR T cells concurrently with on-target, on-tumor efficacy in preclinical mouse models with human tumor xenografts. In comparison, the R6.5 scFv (derived from the mouse hybridoma clone, R6.5^33^) has a K_D_ of 10 nM for human ICAM-1 (Table 1) but does not cross-react with murine ICAM-1.

**Table 1:** Measured affinity of LFA-1 I domain to ICAM-1.

Name	Sequence	Affinity
Wild-type (WT)	G128-G311	1.5 mM*
F292A	G128-G311	20 μM*
F292G	G128-G311	119 nM*
F265S	G128-G311	145 nM*
F265S/F292G (DM)	G128-G311	6 nM*
F265S/F292G (DM)	G128-G311	2 nM^†^
F265S/F292G/G311C (TM)	E124-S313	~1 nM^‡^
R6.5	scFv	10 nM^§^

*SPR measurements of binding to human ICAM-1; ^†^SPR measurements of binding to murine ICAM-1; ^‡^Estimated from soluble human ICAM-1 binding to TM expressing cells by flow cytometry. ^§^Estimated from titrated R6.5 antibody binding to HeLa cells [33]DM, double mutant. TM, triple mutant.

To test whether the mutant I domain affinities correlate with CAR affinities, HEK 293 T and Jurkat T cells were transduced with lentivirus encoding 3^rd^ generation CARs containing TM, F292G, F292A, or WT I domain, and assayed for ICAM-1 binding. A myc tag was appended to the N-terminus of each I domain variant to aid measurement of CAR expression (Fig. 1d,e). To avoid background ICAM-1 binding to endogenous LFA-1 in Jurkat T cells, a relative affinity comparison of CAR variants for ICAM-1 was made using I domain CAR-transduced HEK 293 T cells. The level of recombinant human ICAM-1 binding to I domain CAR-expressing HEK 293 T cells correlated with solution affinity measurements, with TM exhibiting the strongest binding, followed by F292G and F292A, and no detectable binding to WT compared to non-transduced (NT) T cells (Fig. 1f). Differential CAR affinities for ICAM-1 and cross-reactivity with murine ICAM-1 were also examined by measuring cell adhesion to V-bottom plates coated with recombinant human or murine ICAM-1 (Fig. 1g). Jurkat cells transduced with TM and F292G CARs demonstrated a higher level of binding to both human and murine ICAM-1 compared to non-transduced cells. However, despite increased binding of F292A CAR-expressing HEK 293 T cells to soluble ICAM-1 compared to WT I domain-expressing counterparts (Fig. 1f), F292A CAR-Jurkat cells lacked any additional binding to plate-bound ICAM-1 compared to NT or WT I domain-expressing cells (Fig. 1g). In the case of F265S I domain, which demonstrated soluble ICAM-1 binding that was slightly weaker than F292G (145 vs. 119 nM, Table 1), F265S CAR T cells failed to demonstrate any additional binding to plate-bound human ICAM-1 while elevated binding was more apparent to murine ICAM-1. As anticipated, T cells transduced to express R6.5 CAR, which is specific to human ICAM-1 only, exhibited elevated binding to human but not to murine ICAM-1 (Fig. 1g).

### Influence of CAR affinity and target antigen density on CAR T cell activation *in vitro*

**Figure 2:**
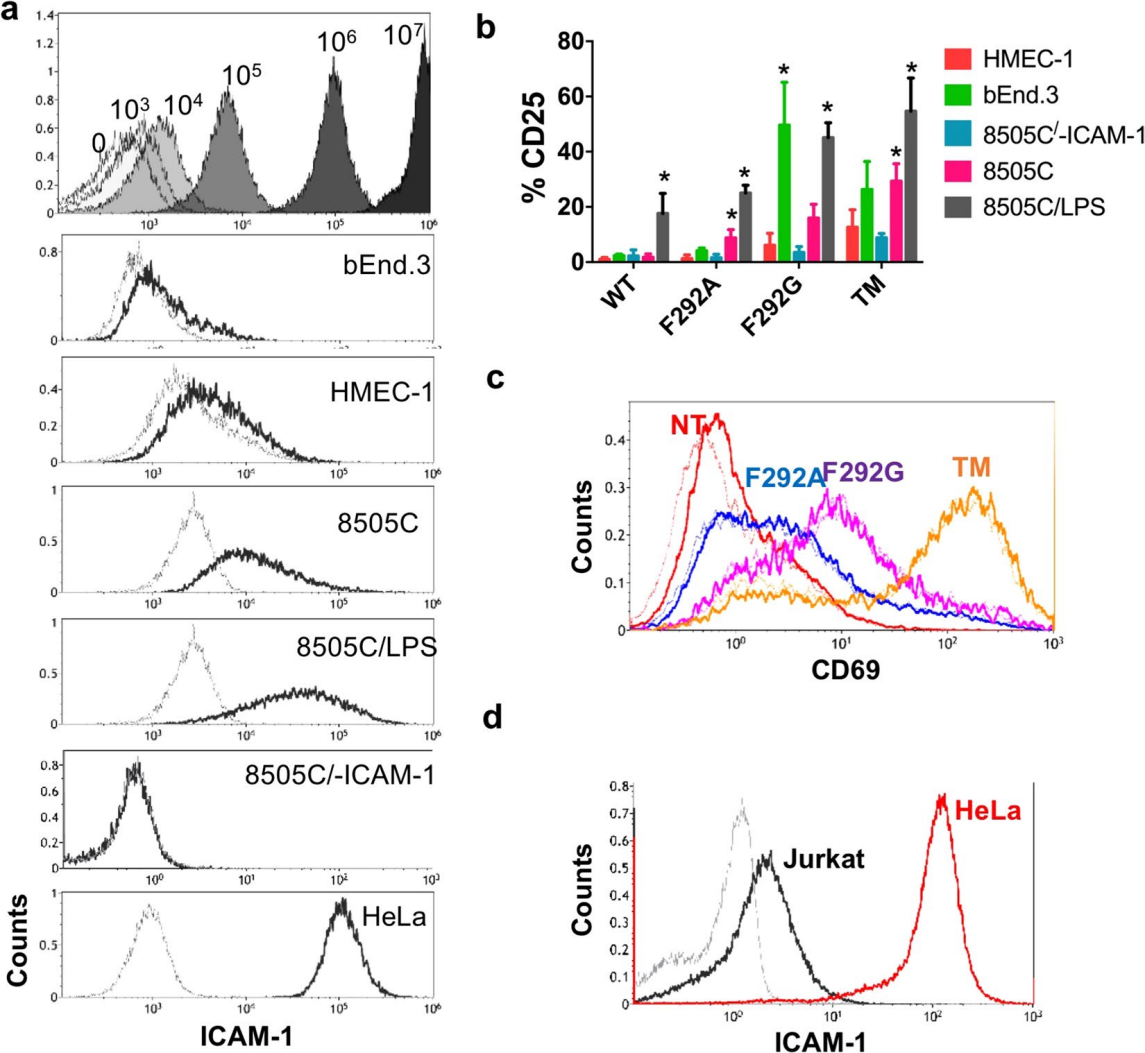
Affinity and antigen-density dependent activation of Jurkat CAR T cells *in vitro*. (**a**) Top, histograms depicting 8 μm latex beads coupled with known amounts of R6.5 antibody conjugated with cy5.5 (10^3^–10^7^ antibodies per bead). The level of shift after incubation with R6.5 (black) from non-labeled (grey) was used to estimate ICAM-1 density in each indicated target cell line. 8505 C/-ICAM-1; 8505 C cells with ICAM-1 gene inactivation by CRISPR/Cas9. 8505 C/LPS, 8505 C cells were incubated with LPS to induce overexpression of ICAM-1. (**b**) CD25 expression in Jurkat CAR T cells (WT, F292A, F292G, and TM) after co-incubation with different target cell lines for 24 h (n = 3–4). *p* < *0*.*01* for * vs. 8505 C/-ICAM-1 by Dunnett’s multiple comparisons test. (**c**) Induction of CD69 after incubation with latex beads coated with 10^6^ recombinant human ICAM-1-Fc molecules. Histograms correspond to CD69 expression in Jurkat cells without (thin, dotted line) and with (thick line) incubation with ICAM-1 coated beads. (**d**) ICAM-1 expression in Jurkat T cells compared to HeLa cells. Grey and black histograms correspond to unlabeled cells and R6.5 antibody-labeled cells, respectively.

Jurkat T cells expressing I domain CARs were used to examine the extent to which CAR T cell activation was influenced by CAR affinity and ICAM-1 antigen density on target cells. Jurkat T cells were incubated with various target cell lines with different ICAM-1 expression levels (Fig. 2a). The surface density of ICAM-1 on target cell lines was first estimated by assaying their levels of anti-ICAM-1 antibody binding and comparing these signals to those obtained using latex beads pre-coated with known amounts of the same antibody (Fig. 2a; top panel). The panel of target cells include: HMEC-1 and bEnd.3, representing, respectively, healthy human and mouse cells with physiological levels of ICAM-1 (~10^4^ molecules per cell); anaplastic thyroid carcinoma (8505 C) expressing an intermediate level (~10^5^ per cell); and cervical cancer (HeLa) cell lines expressing a high level of ICAM-1 (~10^6^ per cell). For additional comparisons, we included 8505 C with CRISPR/Cas9-mediated ICAM-1 gene inactivation (8505 C/-ICAM-1) and 8505 C treated with LPS to upregulate ICAM-1 expression (8505 C/LPS). Activation of CAR T cells upon interaction with target cells was examined by measuring CD25 (IL-2 receptor α) and CD69 expression (Fig. 2b,c). Elevated levels of CD25 were observed in WT I domain CAR T cells following incubation with LPS-stimulated 8505 C but not with other cell lines expressing lower levels of ICAM-1 (Fig. 2b). In contrast, increased CD25 expression was seen when high affinity TM CAR T cells were incubated with high ICAM-1 expressing cells as well as with HMEC-1 and bEnd.3 cells that expressed basal levels of ICAM-1. A low-level of CD25 expression was detected on TM CAR T cells following incubation with target cells lacking ICAM-1 expression (8505 C/-ICAM-1), likely due to homotypic cellular contacts mediated by molecular interactions between TM CAR and basal expression of ICAM-1 on Jurkat cells (~10^4^ molecules/cell; Fig. 2d). T cells expressing F292G behaved similar to TM, except that CD25 expression was close to background levels following co-incubation with 8505 C/-ICAM-1. The micromolar affinity F292A T cells demonstrated selective activation displaying elevated CD25 expression only upon incubation with 8505 C and 8505 C/LPS cells (Fig. 2b). This indicates that a threshold target antigen density of >0^5^ ICAM-1 molecules per cell was required for F292A CAR T cell activation. In contrast to the ICAM-1 density-dependent activation of CD25, increased CD69 expression was observed even in the absence of target cells, with expression levels aligning closely with CAR affinity to ICAM-1, which was not further enhanced by incubation with ICAM-1 coated latex beads (Fig. 2c). Compared to CD25, CD69 induction appeared to require a lower antigen density threshold for activation, which was provided by homotypic interaction between CAR T cells.

### Influence of CAR affinity and target antigen density on CAR T cell cytotoxicity *in vitro*

**Figure 3:**
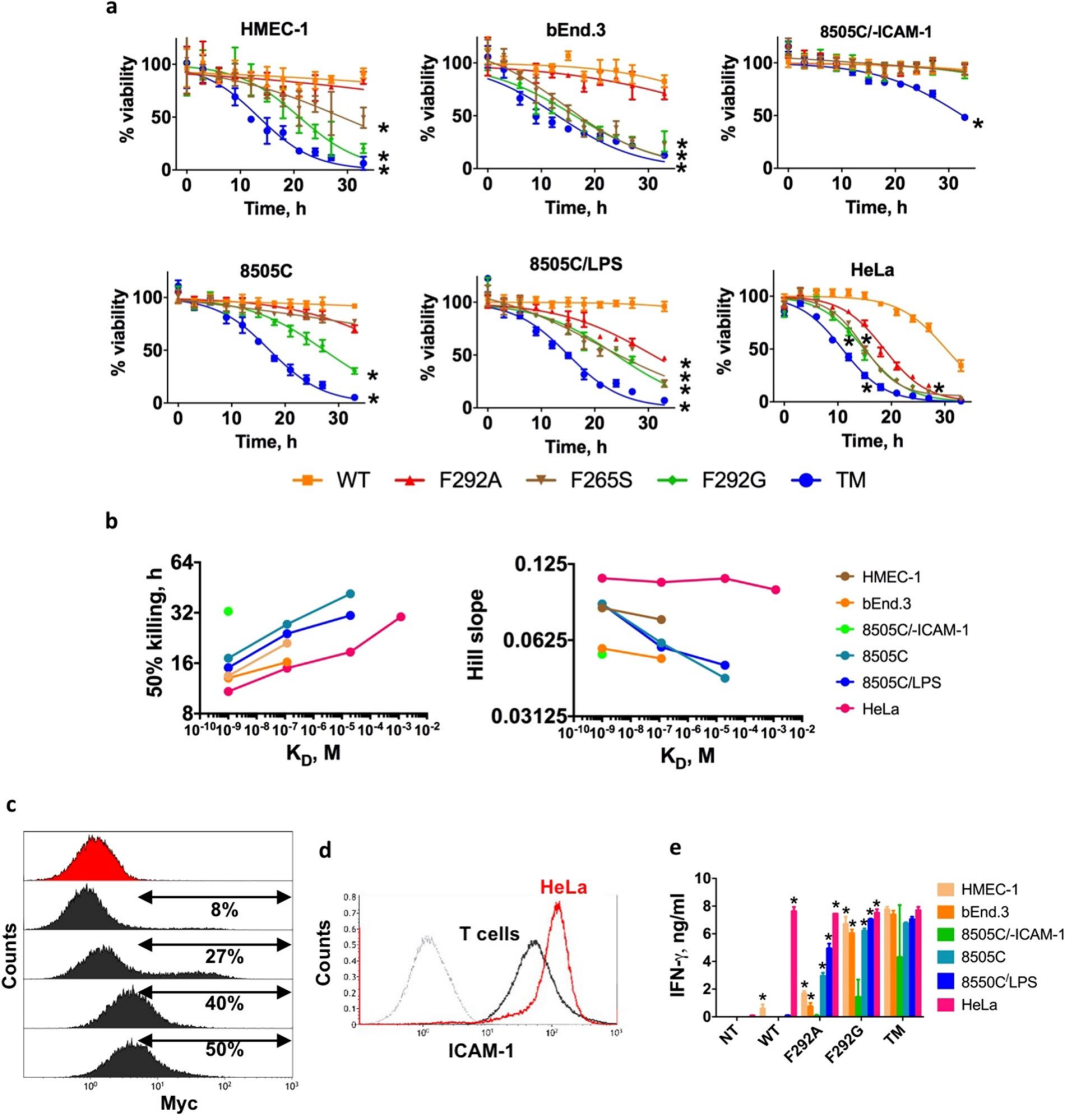
Affinity and antigen-density dependent activation of primary CAR T cells *in vitro*. (**a**) Effector to target (E:T) assay for measuring target killing by primary T cells transduced with different I domain CARs. Each target was separately incubated with TM, F292G, F265S, F292A, or WT CAR T cells at 5:1 E:T ratio. Percent viability was normalized to luminescence from target cells incubated with NT T cells (n = 3, ± = standard deviation (SD)). A variable slope sigmoidal curve equation was used to fit data. *p* < *0*.*01* for * vs. NT by Dunnett’s multiple comparisons test. (**b**) The best fit values of 50% killing and Hill slope of the sigmoidal equation were plotted against the affinities of I domain CARs. The best fit values with r-square values higher than 0.85 were plotted. (**c**) Examples of CAR expression in primary T cells after transduction with different batches of lentivirus or different donor T cells as measured by flow cytometry. Histograms in black for transduced and in red for non-transduced T cells as a control, which were stained with anti-Myc antibody. (**d**) ICAM-1 expression in primary T cells in comparison to HeLa cells. Grey and black histograms correspond to unlabeled T cells and R6.5 antibody-labeled T cells, respectively. (**e**) IFN-γ release was measured by ELISA for each CAR T variant after co-incubation with different target cells for 24 h (n = 3). *p* < *0*.*01* for * vs. 8505 C/-ICAM-1 by Dunnett’s multiple comparisons test.

After validating affinity and antigen-dependent activation of CAR-modified Jurkat T cells, we sought to examine the influence of CAR affinity and antigen density on primary T cell activation and cytotoxicity *in vitro*. Primary T cells were transduced with TM, F292A, F292G, F265S, and WT I domain CARs, and added to various target cells to determine their cytotoxic efficacy *in vitro*. Overall, there was a positive correlation between the rate of target cell lysis and ICAM-1 expression (HeLa > 8505 C/LPS > 8505 C > HMEC-1 > bEND.3) across all I domain variant CAR T cells (Fig. 3a). The rate of killing was also faster when T cells expressed CARs possessing higher affinity for ICAM-1 (TM > F292G > F265S > F292A > WT). To quantitatively compare the efficacy of killing by affinity variant CAR T cells, a variable slope sigmoidal curve (%live = 100/[1 + 10^(t-τ_50%)* Slope^]) was used to find the best fit values describing the time required to achieve 50% killing (τ_50%) and the rate of target killing (Hill slope) (Fig. 3b). The time to 50% target killing was longer with either lower affinity CAR T cells or lower antigen density for the same CAR T cells. Likewise, the Hill slope was higher with increases in affinity (lower K_D_) for the same target cells and increases in antigen density for the same CAR T cells. CAR T cell killing of target cells was specific as evidenced by the lack of observed killing of ICAM-1 negative 8505 C cells by all of the I domain variant CARs except TM. The transduction efficiency of CARs ranged typically between 8–50% with no systematic bias among different CAR variants (Fig. 3c). Low yet gradual killing of 8505 C/-ICAM-1 by TM T cells was likely due to cytotoxic activation caused by homotypic cellular contacts mediated by TM interaction with ICAM-1 in T cells. ICAM-1 expression in primary T cells can be induced after T cell activation^34^ such as by incubation with CD3/CD28 beads (~10^5^ molecules/cell; Fig. 3d). In contrast, WT CAR T cells possessing millimolar affinity (K_D_ = 1.5 mM) could specifically lyse HeLa cells only, indicating a threshold antigen density of approximately 10^6^ molecules per cell for ~1 mM K_D_ CAR T cells. Importantly, F292A and WT I domain CAR T cells (K_D_ > 10 μM) were unreactive to human and murine healthy control cells, HMEC-1 and b.END3 (~10^4^ per cell; Fig. 3a). F265S CAR T cytotoxic activity fell between that of F292A and F292G. It was comparable to F292A against target cells with lower ICAM-1 expression (HMEC-1 and 8505 C) but more similar to F292G against target cells with elevated expression (8505 C/LPS and HeLa). F265S CAR T seemed to react more strongly against murine cells with basal expression of ICAM-1 compared their human counterparts (bEnd.3 vs. HMEC-1), concurring with a higher level of cell adhesion to murine rather than human soluble ICAM-1 (Fig. 1g).

IFN-γ release by CAR T cells aligned closely with the rate of target cell death, where increasing levels were found in co-cultures containing higher affinity CAR T cells and/or higher levels of target antigen expression (Fig. 3e). Exceptions to target antigen density-dependent IFN-γ release were TM and F292G CAR T cells, which produced significant amounts of IFN-γ (>1 ng/ml) in the absence of target molecules (8505 C/-ICAM-1). This is again likely due to the homotypic interactions between T cells, an observation supported by the difficulty of expanding TM CAR T cells, particularly when the level of CAR expression was high. Release of IFN-γ by micromolar affinity CAR T cells (F292A) was proportional to the ICAM-1 density in target cells, demonstrated by a lack of release upon incubation with 8505 C/-ICAM-1, and progressively increasing upon incubation with HMEC-1, 8505 C, 8505 C/LPS, and HeLa in this order (Fig. 3e). Consistent with WT I domain’s cytotoxicity toward HeLa cells, IFN-γ release upon incubation with HeLa was comparable to the levels secreted by other higher affinity CAR T cells.

### *In vivo* efficacy of affinity-tuned I domain CAR T cells

**Figure 4:**
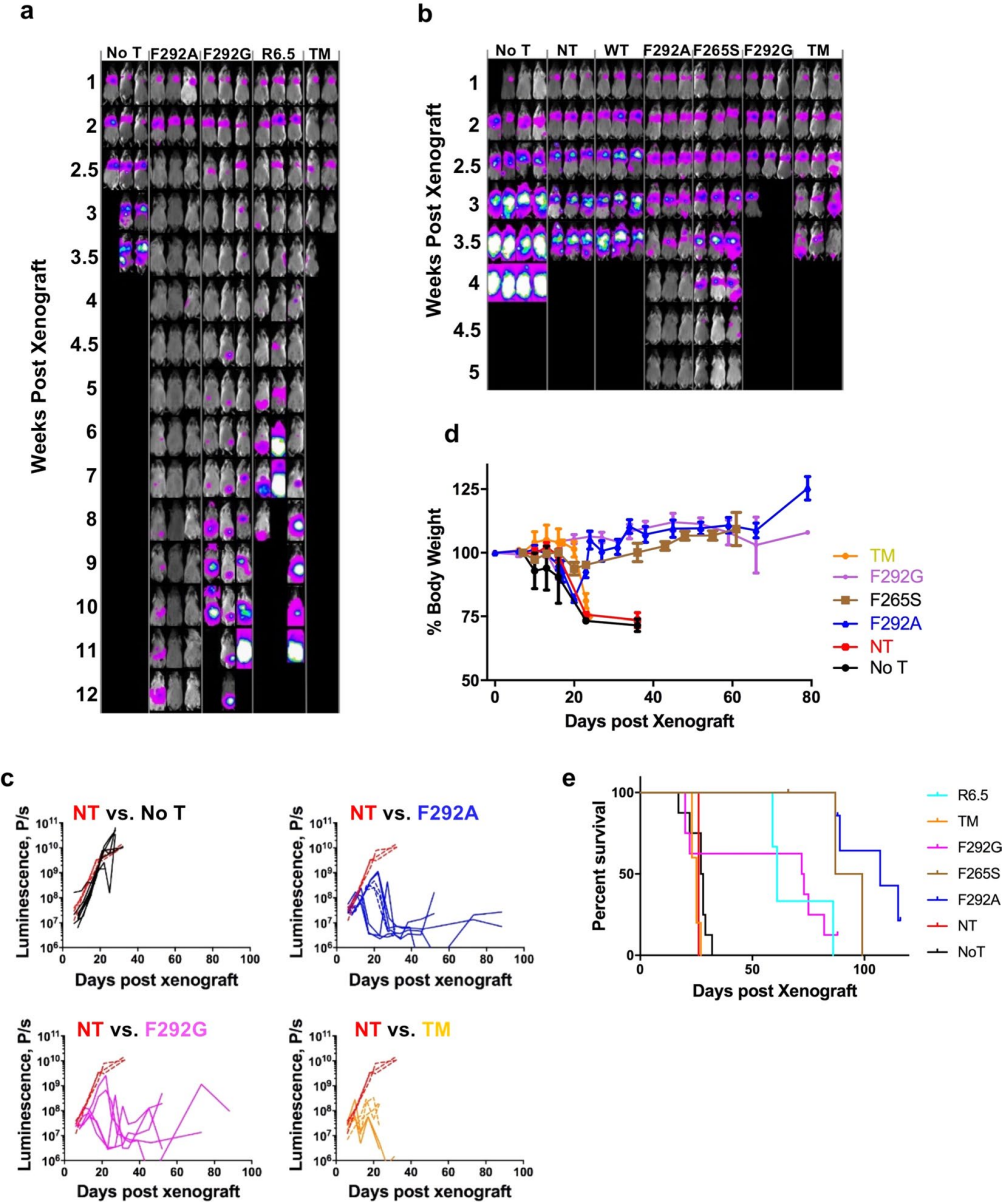
Micromolar affinity CAR T cells provide superior tumor eradication, suppression of tumor relapse, and survival benefit. (**a**) Whole-body luminescence imaging was used to estimate tumor burden in mice infused with different CAR T cell variants 8 days post-tumor implantation. No T = mice received no T cells. (**b**) Mice were treated with CAR T cells 10 days post tumor implantation. NT = non-transduced T cells. (**c**) Luminescence measurements of mice treated with different CAR T cells. Individual lines indicate luminescence values from each mouse. P values of two-tailed student’s t-test versus NT (n = 3) are not-significant for No T (n = 8), p < 0.001 for F292A (n = 8) and F292G (n = 5), and p < 0.05 for TM (n = 5). (**d**) Percentage body weight fluctuation from initial weight on day 0 post tumor xenografts of mice receiving different treatments are depicted. (**e**) Survival curves of mice receiving different treatments. Log-rank (Mantel-Cox) test P values versus NT are not-significant for No T and TM, and p = 0.008 for F292G, p = 0.025 for R6.5, and p = 0.0016 for F292A.

We examined how affinity-dependent CAR T cell cytotoxicity patterns *in vitro* would translate to tumor xenograft models *in vivo*. In solid tumors, CAR T cell efficacy is influenced by their ability to traffic to tumor sites, penetrate, serially lyse tumor cells, and undergo expansion and contraction in accordance with tumor burden. Here, mice were xenografted by systemic *i*.*v*. injections of 0.75 × 10^6^ 8505C-FLuc^+^GFP^+^ cells followed by treatment with ~1–3 × 10^6^ I domain CAR T cells (WT, F292A, F265S, F292G, and TM), SSTR2-R6.5 CAR^29^, NT T cells, and no T cells at 8–10 days post-xenograft. Tumor burden was evaluated by whole-body luminescence imaging of firefly luciferase activity. Primary tumors localized to the lungs and liver with distant metastatic foci evident throughout the body (Fig. 4a). Cohorts receiving either no T cells or NT T cells succumbed to tumor burden within 3–4 weeks of tumor inoculation. Mice treated with TM CAR T cells displayed rapid initial reductions in tumor burden; however, by approximately 7 days post T cell injection, mice began to show symptoms of systemic toxicity indicated by lethargy and weight loss, resulting in death by day 15 post treatment (Fig. 4a–d). F292G CAR T cells were capable of tumor elimination with inconsistent toxicity development, which appeared to be partially dependent on tumor burden at the time of CAR T cell treatment. For example, delayed infusions of F292G CAR T cells (day 10) or higher tumor burden at the time of treatment led to uniform deaths. T cells expressing F265S CAR molecules (Table 1) eliminated tumors without observable toxicity. This suggests that an I domain CAR affinity of ~100 nM K_D_ defines an approximate threshold affinity above which treatment leads to reduced discrimination between high and low antigen densities and an increased likelihood of on-target off-tumor toxicity. Consistent with limited or lack of killing of 8505 C by WT CAR T cells *in vitro*, tumor progression *in vivo* was unimpeded by their infusion for treatment, similar to NT T cells (Fig. 4b). In contrast, F292A CAR T cells, which exhibited a much slower *in vitro* rate of 8505 C killing compared to its higher affinity counterparts, achieved rapid reductions in tumor burden irrespective of treatment timing (Fig. 4a–c). Mice treated with CAR T cells such as F292A and F265S experienced an initial weight loss of up to 20% before rebounding to normal weight after tumor elimination, indicating that there was no immediate or lingering off-tumor toxicity caused by these CAR T cells (Fig. 4d).

Moreover, F292A CAR T *in vivo* efficacy was superior to the scFv-based R6.5 CAR despite an over 1,000-fold lower affinity to ICAM-1 (10 nM vs. 20 µM), as evidenced by a faster rate of tumor clearance and durable suppression of tumor relapse (Fig. 4a). The anti-tumor efficacy of I domain CAR T cells led to statistically significant increases in cohort survival compared with no T or NT T cell-treated mice (Fig. 4e). Overall, the rate of tumor killing or the onset of toxicity *in vivo* were determined primarily by the affinity of CARs, without being noticeably influenced by the level of CAR transduction or expression. However, CAR T cell-treated mice with minimal residual tumor burden began to display signs of toxicity (e.g., weight loss, loss of fur) that typically resulted in eventual death approximately 10 weeks after T cell injection. This was suspected to be related to graft-versus-host disease (GvHD)^35^ and not on-target, off-tumor toxicity as similar toxicities were observed in mice treated with R6.5 CAR T cells that exclusively target human ICAM-1. GvHD-mediated deaths could be distinguished from deaths caused by tumor burden or on-target, off-tumor toxicity by the observation of minimal tumor burden (tumor luminescence < 10^8^ P/s) at the time of death with mortality occurring greater than 70 days post xenograft. Suspected on-target off-tumor toxicity caused by TM or F292G CAR T cells typically occurred within 21 days of T cell infusion. Among deaths that occurred 60 days post xenograft, GvHD accounted for all of F292A CAR T treated mice death (6 out of 6 mice), while GvHD (3 out of 5) and tumor relapse (2 out of 5) were the cause for F292G CAR-treated mice. Tumor relapse was similarly responsible for all R6.5 CAR-treated mice deaths (3 out of 3).

### Cellular analysis of CAR T cell efficacy and toxicity

**Figure 5:**
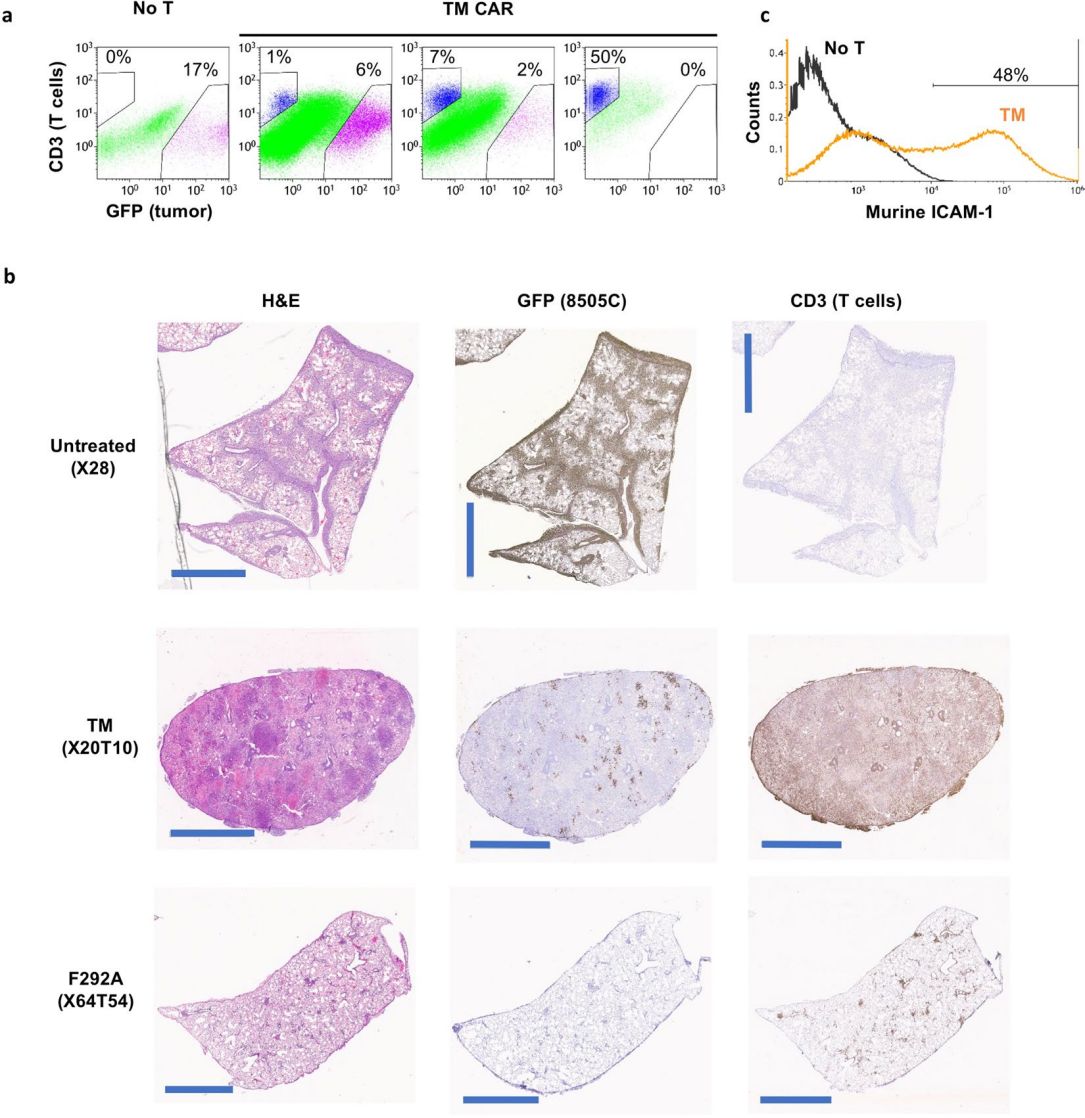
Analysis of systemic toxicity by flow cytometry and histology. (**a**) Flow cytometry analysis of total cells harvested from the lungs of non treated (No T) or treated (TM CAR) mice. GFP and CD3 were used as markers of 8505 C tumor and T cells, respectively. Mice treated with TM CAR T cells were sacrificed at different time points to represent the association between tumor burden and TM CAR T cells. (**b**) Lung tissues harvested from untreated mice and mice treated with TM and F292A CAR T cells were processed for H&E, GFP and CD3 staining. The scale bar in each image is 2 mm. Tissue sacrifice time points are indicated on the left. For example, X20T10 represents 20 days post tumor xenograft and 10 days post T cell infusion. (**c**) Murine ICAM-1 expression in lung cells harvested from tumor-bearing mice after treatment with TM CAR T cells.

*Ex vivo* cellular analysis of tumor cells and T cells by flow cytometry revealed the potential cause for systemic toxicity induced by high affinity CAR T cells, such as TM. Similar to the previously observed biphasic expansion and contraction response of human ICAM-1 specific, R6.5 CAR T cells^29^, TM CAR T cells also exhibited gradual expansion concurrent with reductions in tumor burden. However, in contrast to R6.5 CAR, TM CAR T cells continued to expand even after tumor elimination, eventually accounting for approximately 50% of live cells in the lungs at the time of death or sacrifice (Fig. 5a). This expansion of TM CAR T cells in the absence of, or in the presence of negligible tumor burden, was confirmed by histology (Fig. 5b). Compared to the heavy tumor burden in lungs of untreated mice, the lungs from mice treated with TM CAR T cells exhibited sparsely distributed tumor cells while demonstrating concurrent heavy infiltration with human CD3 positive T cells. In comparison, the lungs of mice treated with F292A CAR T cells were almost entirely tumor-free with only sparsely distributed T cells present, indicative of the expected T cell contraction that was previously observed with R6.5 CAR T cells after tumor elimination in the same model^29^. The near uniform systemic toxicity observed in mice treated with TM CAR T cells appeared to depend upon the initial presence of tumor as there was no apparent systemic toxicity observed in non-tumor bearing mice that received TM CAR T cells (4/4 mice surviving more than 2 months after T cell infusion). We speculate that TM and at times, F292G CAR T cells reacted with murine ICAM-1 that was upregulated in response to exposure to inflammatory cytokines secreted by CAR T cells or lysed tumor cells during engagement with human ICAM-1-expressing tumors. Indeed, similar to the induction of ICAM-1 expression in murine lungs by LPS in our previous study^36^, around 50% of live lung cells from TM CAR T-treated mice stained positive for murine ICAM-1 (Fig. 5c), compared to much lower expression and percentage of ICAM-1 stained cells from non-tumor bearing mice (<5% in untreated lungs vs. >50% after LPS treatment, Fig. 6 a, b in Kang *et al*.^36^), or tumor-xenografted mice without T cell treatment (<5%, Fig. 5c).

**Figure 6:**
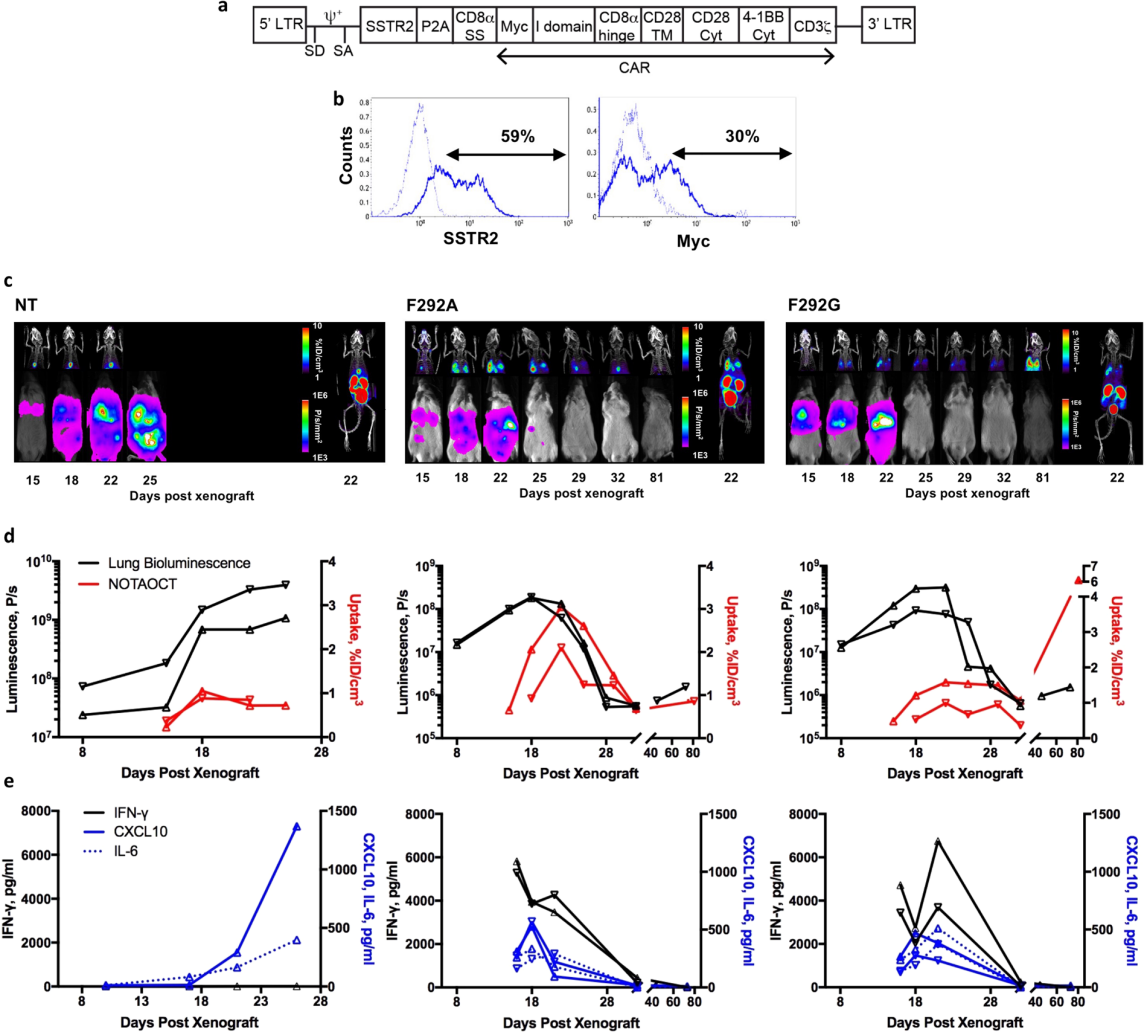
Longitudinal, concurrent measurements of tumor burden, T cell distribution, and cytokine release. (**a**) Schematic of SSTR2-I domain vector. (**b**) Flow cytometry measurements of SSTR2 reporter gene and Myc-tag expression representing CAR on the surface of primary human T cells. Thin and thick histograms represent T cells with and without antibody labeling, respectively. (**c**) Longitudinal measurements of NOTAOCT uptake by PET/CT (top half of each panel), and tumor burden by whole body luminescence imaging (bottom half of each panel). Images are representative of 2–4 mice in each cohort. Whole body PET/CT images, taken on the day of maximum tracer uptake, are shown on the far right. Imaging time points are indicated below the bottom panel. (**d**) Quantification of luminescence and tracer uptake in the lungs of two mice in each cohort. (**e**) Cytokine levels measured from blood drawn at various time points from the matching mice in ‘d’ are plotted.

### Real-time imaging of CAR T cell kinetics, efficacy, toxicity, and correlation with cytokine profiles

To spatiotemporally monitor T cell distribution in real-time by PET/CT, we introduced an imaging reporter gene, SSTR2, into the I domain CAR vector using a ribosome skipping P2A sequence to ensure equal expression of CAR and the reporter on the surface of T cells (Fig. 6a). Expression of SSTR2 enables binding and intracellular accumulation of an infused, positron-emitting, SSTR2-specfic radiotracer, ^18^F-NOTA-Octreotide (NOTAOCT)^30^. Emitted signals can then be detected with high resolution without limits on tissue penetration by a micro PET scanner. Expression of SSTR2 and Myc-tagged I domain was confirmed by antibody staining, although a higher level of binding was found with the SSTR2 antibody, likely due to lower background noise (Fig. 6b). Mice were xenografted with 8505 C tumors as before, and were treated with NT, F292A, or F292G CAR T cells 8 days post tumor xenograft. Whole-body luminescence imaging was performed to estimate tumor burden while PET/CT imaging was performed on the same day to track CAR T cell distribution (Fig. 6c). At selected imaging time points, blood was collected within 24 h to measure human cytokines for correlation with observed T cell dynamics. PET/CT images in mice displayed expected background uptake levels of NOTAOCT in gall bladder, kidneys, and bladder caused by radiotracer excretion^31,37^ (Fig. 6c; far-right). In the NT treated control cohort, a small but gradual increase in non-specific tracer uptake was observed, which was due to increasing tumor burden and the associated increase in blood pooling (Fig. 6c,d). In contrast, specific tracer uptake was observed in mice treated with SSTR2-F292A CAR T cells, demonstrating the expansion and contraction phases in the lungs, with peak CAR T cell signal occurring approximately 4 days following peak tumor burden and gradually decreasing to background levels (Fig. 6c,d). This biphasic T cell expansion and contraction phenomenon was reminiscent of our previous study using SSTR2-R6.5 CAR T cells in the same mouse tumor model^29^. Cytokine analysis of serum obtained from treated mice demonstrated a surge in IFN-γ, IL-6, and CXCL10 concentrations prior to peak T cell expansion, which also returned to background levels post tumor elimination and following contraction of T cell density in the lungs to background levels (Fig. 6e). F292G CAR T-treated mice displayed a similar yet lower T cell expansion profile in the lungs. Here, the serum cytokine profile was similar to that observed in F292A CAR T-treated mice. In some instances, treatment with F292G CAR T led to host mortality, likely due to on-target, off-tumor toxicity mediated by CAR T cells. This cause was postulated due to an unexpected dramatic increase in F292G CAR T cells in the lungs, as detected by PET/CT imaging, long after tumor elimination and CAR T cell contraction (Fig. 6d). Based on our previous study, we estimate that a tracer uptake of approximately 6% ID/cm^3^ corresponds to a T cell density in lungs above 10% of all cells (equivalent to ~3% ID/cm^3^ in 10% SSTR2 positive Jurkat mosaic tumors^29^). This level of tracer uptake was higher than the initial tumor-dependent T cell peak and was similar to that observed during TM CAR T cell treatment, where T cells accounted for > 50% of total cells in lungs post tumor eradication (Fig. 5a). This high level of CAR T expansion in tumor-free lungs was not observed in mice treated with F292A CAR T cells even with an emerging sign of GvHD (tracer uptake < 1% ID/cm^3^, e.g., F292A in Fig. 5d).

## Discussion

The paucity of native, surface-expressed, and tumor-specific antigens presents a significant hurdle to developing CAR T cell therapy against solid cancers^38,39^. Conventional CARs are constructed using a single-chain antibody format, and are selectively engineered to possess sub- to low nanomolar affinities for target antigens. However, increased CAR T cell sensitivity may be an advantage only when targeting true tumor antigens or those with the highest levels of restriction^17,38^. Otherwise, increased sensitivity comes at the price of reduced selectivity with lysis of target-expressing cells occurring in a manner largely insensitive to antigen density^18^. By functionally investigating CAR affinities spanning step-wise across a 10^6^-fold range, concurrently targeting cells with varying levels of antigen expression, we systematically examined the influence of CAR affinity and antigen density on T cell efficacy *in vitro* and *in vivo*. T cell activation status *in vitro*, as measured by CD25, cytokine release, and cytotoxicity, was to a remarkable degree dependent on affinity and target antigen density, resulting in more potent T cell activation and target killing with increases in CAR affinity and antigen density. The activation threshold of nanomolar affinity CAR T cells (TM, F292G) was less dependent on antigen density compared to micromolar affinity CAR T cells (F292A), reacting to antigen densities as low as 10^4^ molecules/cell. In contrast, F292A CAR T cells rapidly lost the ability to lyse cells expressing target antigens below 10^5^ molecules/cell. Millimolar affinity CAR T cells (WT) were largely unreactive to target cells with low to moderate levels of antigen, requiring a threshold antigen density of 10^6^ molecules/cell for detectable activation, cytokine release, and target lysis to occur. The quantitative harmony between CAR affinity and anti-tumor potency *in vitro* was discordant with quantitative *in vivo* observations whereby micromolar affinity CAR T cells were superior to higher affinity CAR T cells as measured by the rate of expansion at the tumor site, the rate of tumor eradication, frequency of tumor relapse, and levels of on-target, off-tumor toxicity.

The ability of I domain CAR T cells to cross-react with murine ICAM-1 allowed for a simultaneous and rigorous assessment of CAR T cell efficacy against human tumor cells and on-target, off-tumor toxicity against murine ICAM-1 on healthy tissues. Onset of toxicity appeared to be dependent on CAR affinity and tumor-burden, as demonstrated by the uniform fatalities in mice treated with the highest affinity (TM) CAR T cells, the increased rate of toxicity observed in F292G CAR-treated mice with larger tumor burden, and the absence of detectable toxicity after treatment with micromolar affinity F292A CAR T cells. The relationship between tumor-burden and CAR-mediated toxicity is likely a consequence of ICAM-1 biology. ICAM-1 is an inducible adhesion molecule that is sensitive to inflammatory stimuli and the tumor-related inflammatory milieu^40–43^. We suspect that cytokine-induced ICAM-1 overexpression is a reason for the onset of toxicity mediated by the high affinity CAR T cells, as demonstrated by the lack of apparent toxicity following TM CAR T treatment in non-tumor bearing animals. This observation suggests that either the basal ICAM-1 density is below the threshold antigen density for TM (10^4^ molecules/cell) or that cells expressing ICAM-1 above this threshold are not exposed to TM CAR T cells. Leukocyte adhesion and diapedesis requires LFA-1 to be in an activated, open configuration along with concurrent upregulation of ICAM-1 on endothelial cells^44^. This augments the hypothesis that ICAM-1 upregulation on healthy tissues is also necessary for activation of even nanomolar affinity I domain CAR T cells. Rapid expansion of CAR T cells in response to growing tumor burden may drive local concentrations of inflammatory cytokines to breach the antigen threshold to cause both F292G and TM CAR T cells to become fatal to the host despite the approximately 100-fold difference in affinity. Despite induction of similar peripheral concentrations of inflammatory cytokines (IFN-γ, IL-6, and CXCL10) in mice treated with F292G or F292A CAR T cells, local or systemic toxicity by F292A CAR T was not observed. Given the estimated threshold antigen density requirement of ~10^5^/cell for F292A mediated-cytotoxicity to occur, we speculated that inflammation-induced murine ICAM-1 may still be below this threshold. Systemic toxicity and fatalities were also absent in animals treated with F265S CAR T cells. The F265S CAR demonstrated weaker binding to plate-bound ICAM-1 *in vitro* compared to F292G CAR despite their comparable solution affinity to ICAM-1.

To investigate the dynamics of affinity-variant CARs *in vivo*, we employed longitudinal concurrent measurements of cytokine release, T cell distribution, and tumor killing. In our model, high concentrations of IFN-γ, IL-6, and CXCL10 were detected as early as 7 days post-infusion of T cells, indicating that T cells were engaging their target and proliferating. In agreement with our previous study using R6.5 CAR T cells targeting human ICAM-1 only^29^, there was a time delay between peak tumor burden and peak I domain CAR T cell expansion as measured by PET/CT. The swift reduction in tumor burden and CAR T cell numbers at the tumor site, along with the resolution of inflammation as measured by cytokines, is indicative of therapeutic efficacy in a manner similar to classical infection scenarios^45,46^. This biphasic response was found to be absent in situations where CAR T cells fail to properly control tumor growth^29^. The onset of cytokine release detected prior to observable T cell expansion by PET/CT using the SSTR2 radiotracer suggests that any improvement in imaging sensitivity (currently 1% SSTR2^+^ T cell density within tumors) can shorten this imaging latency period and aid in determining if heightened T cell activity is at on- or off-target sites. Peak serum IL-2 levels were determined to be relatively low (<50 pg/ml) in comparison to IFN-γ, IL-6, and CXCL10 levels. As severe cytokine release syndrome was suspected as the cause of death in some patients enrolled in CD19-targeting CAR T cell trials, our cytokine kinetics data suggests that blood analysis immediately after T cell infusion will be critical to estimate early T cell activity. Upon observation of a cytokine surge, patients receiving tumor-targeted T cells expressing SSTR2 could be imaged via PET/CT to examine the T cell distribution profile and determine whether they are expanding at the tumor site, or, if they are expanding away from the tumor, thus indicating the onset of off-tumor toxicities. In our study, the continued expansion of the nanomolar affinity F292G CAR T cells despite tumor elimination was likely caused by activation of CAR T cells by inflammation-induced murine ICAM-1. Such instances of off-tumor T cell expansion in patients could be easily diagnosed and localized by imaging techniques such as PET/CT, which we anticipate will be increasingly utilized in future adoptive T cell trials. The provision of detailed, real-time T cell trafficking information would aid general therapy development including dosing protocols and intervention strategies to avoid harmful or even fatal toxicities by administered cells^47,48^.

The functional goal when creating affinity-tuned CAR T cells in prior studies has been to minimize off-tumor toxicity against basally expressed antigens in normal tissues^18,49^. However, we believe that CARs possessing affinities in the micromolar range may be superior to those in the nanomolar range for additional reasons. Engagement of target antigen by nanomolar affinity CAR T cells (e.g., TM, F292G, and R6.5 CAR) likely results in an unnaturally slow off rate, deviating from transient and dynamic nature of interactions natively found between TCRs and pMHCs^50^. High affinity and avidity interactions by CAR can reduce T cells’ propensity for serial killing, potentially causing exhaustion or increased susceptibility to activation-induced cell death^51^. The fact that the majority of CARs developed to-date rely upon nanomolar-affinity scFvs raises the possibility that these CAR T cells may not achieve their maximum potential therapeutic index. This speculation also draws on prior observations on the influence of high-affinity TCR and pMHC interactions on T cell fate^15,16,21,23,52^. Future CAR engineering strategies should evaluate a broader affinity range to examine the influence on T cell behavior and functionality *in vivo* more rigorously. This particularly applies to CARs targeting solid tumor antigens, which are likely to require evolved strategies to counter the more challenging environment.

In summary, our study demonstrates that CAR T cells with target-affinities mimicking that of native TCR for pMHC can eschew targeting of healthy tissue with basal antigen expression while simultaneously exhibiting increased potency and long-term efficacy against tumor tissue with high target expression. The micromolar affinity F292A CAR enabled T cells to neglect tissues expressing less than 10^5^ molecules/cell, a threshold which anaplastic thyroid tumors surpass yet healthy tissues, that we examined, do not. Various protein engineering approaches including directed evolution, alanine substitution within antibody complementary determining regions, or humanization of mouse antibodies^27,49,53,54^ to isolate lower affinity variants can be used to modulate the affinity of scFv-based CARs or TCRs. Systematic studies examining the influence of scFv-based CAR (e.g., CD19 CAR) affinity on CAR T cell efficacy and toxicity will be necessary to further examine the hypothesis that T cells expressing traditional nanomolar affinity CARs are operating sub-optimally and may be more prone to exhaustion and excessive cytokine release, ultimately facilitating off-tumor toxicity or tumor relapse.
